# TAD cliques predict key features of chromatin organization

**DOI:** 10.1186/s12864-021-07815-8

**Published:** 2021-07-03

**Authors:** Tharvesh M. Liyakat Ali, Annaël Brunet, Philippe Collas, Jonas Paulsen

**Affiliations:** 1 Department of Molecular Medicine, Institute of Basic Medical Sciences, Faculty of Medicine, University of Oslo, Oslo, Norway; 2 Department of Immunology and Transfusion Medicine, Oslo University Hospital, Oslo, Norway; 3 Institute of Biosciences, Faculty of Mathematics and Natural Sciences, University of Oslo, Oslo, Norway

**Keywords:** 3D genome, chromatin conformation, Hi-C, TAD, CTCF motif

## Abstract

**Background:**

Mechanisms underlying genome 3D organization and domain formation in the mammalian nucleus are not completely understood. Multiple processes such as transcriptional compartmentalization, DNA loop extrusion and interactions with the nuclear lamina dynamically act on chromatin at multiple levels. Here, we explore long-range interaction patterns between topologically associated domains (TADs) in several cell types.

**Results:**

We find that TAD long-range interactions are connected to many key features of chromatin organization, including open and closed compartments, compaction and loop extrusion processes. Domains that form large TAD cliques tend to be repressive across cell types, when comparing gene expression, LINE/SINE repeat content and chromatin subcompartments. Further, TADs in large cliques are larger in genomic size, less dense and depleted of convergent CTCF motifs, in contrast to smaller and denser TADs formed by a loop extrusion process.

**Conclusions:**

Our results shed light on the organizational principles that govern repressive and active domains in the human genome.

**Supplementary Information:**

The online version contains supplementary material available at 10.1186/s12864-021-07815-8.

## Background

Spatial organization and packaging of the genome are important for proper regulation of gene expression and are often altered in disease [1]. Understanding the underlying organizational principles of 3D genome architecture requires a multi-scale and multi-scope approach. At higher-order levels, chromosomes seem to organize into two large A and B compartments which can be computed from the first eigenvector of a principal component analysis of a correlation Hi-C matrix at low resolution (e.g. 1 megabase [Mb]) [2]. By definition, A compartments constitute open/active parts of the genome, while B compartments make up the remaining inactive parts. Increasing resolution, thus decreasing the bin size of a Hi-C matrix, reveals a finer delineation of compartments into subcompartments [3]. Zooming further on the diagonal of the Hi-C matrix reveals nested levels of high-frequency interactions delineated by relatively abrupt boundaries between them, referred to as topologically-associated domains (TADs) [4, 5]. Several processes together likely shape the chromosomal interaction patterns observed in Hi-C matrices. Phase separation has been proposed to explain the formation of heterochromatin compartments [6, 7], and a loop extrusion model could explain TAD formation and dynamics [8, 9]. For most genomic regions, multiple processes act simultaneously within and between cells in a population to spatially organize the genome at multiple levels [10–12].

Based on analysis of the *Drosophila* genome, high-resolution Hi-C data show that compartments of very small sizes can be computed from an eigenvector analysis similar to what has previously been applied on low-resolution Hi-C data [13]. These compartments, termed compartment domains, correspond almost perfectly to transcription state transitions in the *Drosophila* genome [13]. Such compartment domains are also found in mammalian genomes [13]. However in addition, chromatin looping events involving CCCTC-binding factor (CTCF) seem to play a prominent role in the formation of TADs [3], in particular through loop extrusion processes [8, 9]. Simulations reveal that small compartment domains are partially suppressed by loop extrusion processes counteracting their segregation [10]. The view of mammalian 3D genome organization is thus becoming increasingly complex, and further classification of the various types of chromatin domains has been suggested [14].

An emerging strategy to model ChIA-PET or Hi-C data entails using graph-based approaches. These have been utilized to establish functional long-range chromatin interaction networks [15–17], and to unravel TAD and sub-TAD structure and their nested hierarchies [18]. Graph-based approaches have also enabled modeling of TAD networks explaining the synchrony of replication timing over long genomic distances [19], and modeling network architecture within TADs to demonstrate that a subset of TADs are structured as core-periphery networks [20]. These networks are interestingly shown to be partially disrupted upon altered CTCF protein levels [20]. We have also recently shown that long-range TAD-TAD interactions can occur in the form of TAD cliques, which we have defined as an assembly of ≥ 3 TADs that are fully connected pairwise in a graph representation of the Hi-C data [21]. TAD cliques associate with key organizational processes during stem cell differentiation, notably by stabilizing heterochromatin at the nuclear periphery, through lamina-associated domains (LADs) [21]. Here, we explore the properties of TADs engaging in TAD-TAD interactions in four differentiated human cell lines. We find that TADs that belong to large or small cliques display distinct genomic features. Most significantly, TADs in large cliques are depleted of convergent CTCF motifs at their boundaries, unlike ‘classical’ TADs explained by chromatin loop extrusion processes. Our findings shed further light on long-range TAD-TAD interactions and indicate that they constitute an important structural feature of the genome.

## Results

**Fig. 1:**
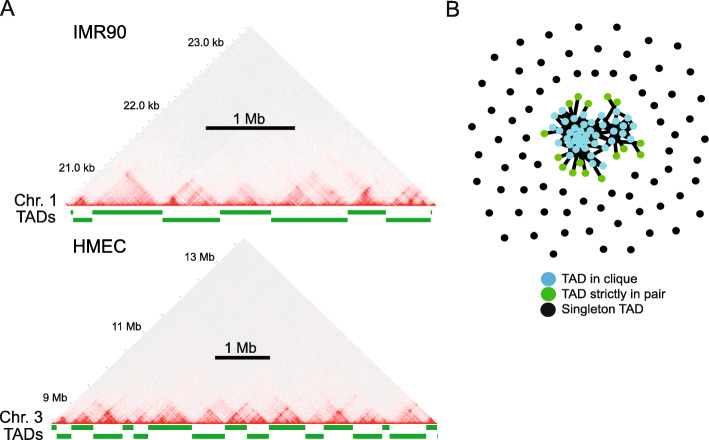
TADs and TAD interaction networks. **(A)** Examples of TADs identified in Hi-C matrices of IMR90 and HMEC cells. Delineation of Armatus TADs is shown as green bars. **(B)** TAD networks: graph representation of TADs in clique, binary-interacting TADs (TADs in pairs only) and singleton TADs for chromosome 18 in IMR90 cells

Long-range interactions between linearly non-contiguous TADs, together with interactions between TADs and the nuclear lamina via LADs, shape genome architecture during differentiation of adipose stem cells [21]. To further explore such TAD-TAD interactions in other, more differentiated, cell types, we analyzed TADs in four human cell lines (HMEC, a mammary epithelial cell line; HUVEC, an umbilical vein endothelial cell line; IMR90, an embryonic lung fibroblast cell line; and K562, an erythroleukemia cell line) for which high-resolution Hi-C and gene expression information is available [22] (see Additional file 1, Table S1 for accession numbers). Using Armatus [23] (see Methods), we identified a total of 5502–6008 TADs in each cell line (Additional file 1, Table S2), consistent with our previous findings in primary human adipose stem cells using the same algorithm [21]. These TADs display similar characteristics as shown earlier [4, 5, 21], with marked boundary structures and sizes in the range of 0.2 to 1 Mb (Fig. 1 A).

### TAD-TAD interactions, TAD cliques and gene repression

To identify TAD-TAD interactions from Hi-C data in HMEC, HUVEC, IMR90 and K562 cells, we used the Non-central Hypergeometric model as done previously [21, 24, 25]. This statistical model takes into account the general propensity for any pair of TADs to engage in contacts, and the genomic distance between them (see Methods for details). We find a total of 5934–8300 significant intra-chromosomal interactions (IMR90: 8300; HMEC: 7309; HUVEC: 5934; K562: 7823). Interactions between TADs are configured as complex networks of strictly pairwise interactions, or involving multiple interactions, with enrichments and depletions of contacts across chromosomes, as exemplified for chromosome 18 in IMR90 cells (Fig. 1B).

**Fig. 2:**
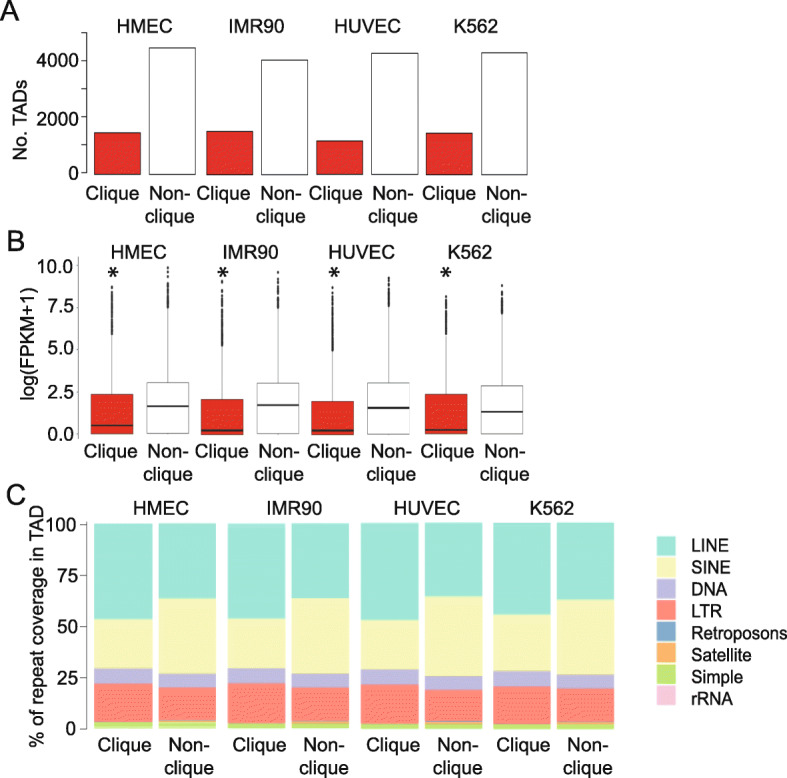
Genomic characterization of TADs in cliques. **(A)** Number of TADs (Armatus) in cliques and outside cliques in indicated cell types, identified from publicly available Hi-C data. **(B)** Gene expression levels in TADs in cliques and outside cliques. *P < 2.2e-16 (Kolmogorov-Smirnov test) compared to non-cliques. **(C)** Proportion of TAD coverage by indicated repeat classes in cliques and outside cliques

TADs can engage in interactions with multiple TADs, some forming cliques (where all TADs interact pairwise [21]), some not. In addition, a TAD can be part of one or more cliques of different size (the size of a clique is defined by the number of TADs that comprise it). We use the term ‘TAD maximal clique size’ when referring to the size of the largest clique a given TAD belongs to [21]. Maximal clique sizes were determined for all four cell types, as done previously using the Bron-Kerbosch algorithm [21]. We find that across cell lines, 1189–1554 TADs engage in associations with at least two other linearly non-contiguous TADs, forming cliques of size ≥ 3 (Fig. 2 A; Additional file 1, Table S2). This represents 21–27 % of all TADs in these cell lines (Fig. 2 A), supporting the view that TAD cliques constitute a significant feature of higher-order genome topology. As previously reported [21], genes residing within TADs in cliques are expressed at a lower level than those in TADs outside cliques (Fig. 2B), corroborating the repressive nature of TAD cliques (see Additional file 1, Fig. S1 for an example of a TAD clique). Gene ontology analyses show that TAD cliques are generally enriched in genes involved in signaling and transcription regulation, indicating that these genes serve important functions (Additional file 1, Fig. S2-S6).

To investigate whether our findings could originate from a mere enrichment of TAD cliques in B compartments, we redid analyses with TADs located exclusively in B compartments. As shown in Additional file 1, Fig. S7A,B, in B compartment specifically, TADs in cliques display similar gene repression properties relative to TADs outside cliques.

Retrotransposons play an increasingly appreciated role in gene expression and chromatin structure regulation [26, 27]. Evidence that long interspersed elements (LINEs) and short interspersed elements (SINEs) can modulate transcription by altering chromatin composition [28] and structure [29] illustrates these elements’ relevance for genome architecture. Notably, LINEs and SINEs may act as euchromatin-heterochromatin boundary elements confining gene expression to the proper compartment [29] or play a role in the formation of silent domains [30]. Analyses of association between LINEs and SINEs and nuclear architecture has also recently suggested that these elements majorly contribute to 3D genome segregation [31]. The relationship between retrotransposons and long-range TAD-TAD interactions has however not been thoroughly examined. We investigated the genomic distribution of repeat classes across TADs in and outside cliques. We find a systematic enrichment of LINE coverage, and correspondingly a depletion of SINE coverage, for TADs in cliques compared to TADs outside cliques (Fig. 2 C). Other repeat classes show limited if any differential coverage (Fig. 2 C). This is also consistent for TADs in cliques specifically within B compartments (Additional file 1, Fig. S7C). As LINE elements are implicated in heterochromatin formation [30], this finding further establishes TAD cliques as repressive sub-compartments of the genome. Of note, enrichment of LINES in TAD cliques is counter-balanced by a depletion of SINEs. Thus, we did not expect a bias in reference genome mappability due to repetitive elements for TADs specifically in cliques. To further investigate possible mappability effects, we intersected our TADs with mappability tracks generated from ENCODE. These results show a stably low (0.1–0.2 %) fraction of base-pairs overlapping such regions (Additional file 1, Fig. S8).

### Genomic characterization of TADs in cliques

As TADs usually are defined solely from short-range Hi-C contact enrichments separated by sharp boundaries [4, 5], the processes underlying their formation could vary between different TADs. Several partially independent processes have been proposed to shape TADs [11, 14]. Loop extrusion has been proposed as an underlying process in TAD formation [8, 9], whereas phase separation has been suggested as a mechanism of compartmentalization of chromatin [6, 7]. In the human genome, a combination of these processes seems to underline the delineation of many TADs [13].

**Fig. 3:**
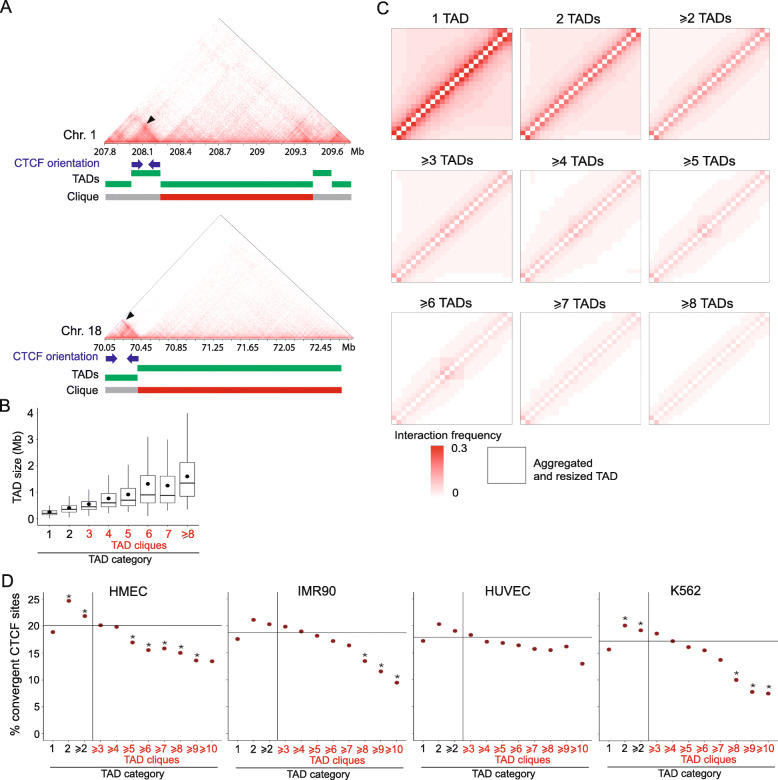
TADs in cliques display less dense interaction patterns than singleton or binary-interacting TADs and are impoverished in convergent CTCF motifs. **(A)** Hi-C matrices for segments of chromosomes 1 and 18 (IMR90 cells); Armatus TADs are delineated by green bars. A TAD belonging to a clique is indicated by a red bar (gray otherwise). Small TADs containing dense chromosomal interactions display convergent CTCF motifs at their boundaries (blue arrows); arrowheads in matrices point to a corner interaction peak. **(B)** TAD size distribution in IMR90 cells as a function of TAD clique size (3 to ≥ 8). Bar, median; dot, mean. **(C)** Aggregation heatmaps showing mean interaction frequencies inside TADs for increasing TAD clique sizes. Each matrix shows the aggregated intra-TAD contacts patterns in the indicated TAD categories (see Methods for details). **(D)** Percentage of convergent CTCF motifs at the boundaries of TADs categorized as shown. The horizontal bar represents the average percentage of convergent CTCF motifs in all TADs genome-wide. *Binomial test; see Table S3 for statistics

Visualization of Hi-C contact patterns within TADs in cliques reveals a distinct contact feature often characterized by larger and less densely interacting domains compared to TADs not in cliques (exemplified in Fig. 3 A). To investigate this further, we determined the distribution of TAD sizes for TADs identified as singletons, TADs interacting only in pairs (binary interacting TADs), and TADs belonging to cliques of increasing sizes. At the whole genome level, we note a linear relationship between clique size and median size of TADs in these cliques (Fig. 3B). Further, genome-wide analysis of Hi-C contact densities within TADs in varying TAD clique classes indicates that TADs in larger cliques systematically display a less dense contact pattern than singleton and binary interacting TADs (Fig. 3 C). The apparent depletion of contacts inside TADs belonging to larger cliques could result from the fact that TADs in large cliques engage in long-range interactions between TADs at the expense of Hi-C interactions occurring within TADs.

The presence and orientation of CTCF motifs at each TAD boundary is indicative of TAD formation and stability [3, 32]. Given our previous observation of higher density interactions within small TADs than in large TADs, we explored the enrichment of convergent CTCF motifs at the boundaries of TADs in the cell lines examined in our study. Interestingly, convergent CTCF motifs and corner peaks seem less prominent for TADs in cliques than for TADs not in cliques (Fig. 3 A, blue arrows and black arrowheads). We therefore hypothesized that the process shaping TADs in cliques might be distinct from that shaping TADs outside cliques.

To test this hypothesis, we computed genome-wide enrichment scores of convergent CTCF motifs for (i) singleton TADs, (ii) TADs involved in strictly binary interactions and (iii) TADs in cliques of increasing size (Fig. 3D). We find that TADs engaging in interaction with only one other TAD are the most enriched in convergent CTCF motifs at their boundaries, whereas TAD in cliques of increasing size show a gradual decrease in convergent CTCF motif enrichment (Fig. 3D). In fact, in large cliques (≥ 5 TADs), convergent CTCF motifs are depleted compared to the average convergent CTCF motif enrichment across all TADs in the genome. For cliques of ≥ 5–8 TADs in HMEC, IMR90 and K562 cells, this depletion is statistically significant (Additional file 1, Table S3). Singleton TADs are less enriched in convergent CTCF motifs than binary interacting TADs, and also depleted compared to the genome-wide average (Fig. 3D). These trends are systematic across the four cell lines, suggesting a general relationship. We note however that for TADs specific to B compartments, similar trends are observed albeit with less or no significance, likely due to the lower number of TADs in each category (Additional file 1, Fig. S9; Table S4).

Since convergent CTCF motifs are implicated in loop extrusion, our data suggest that TADs with few interactions with other TADs are more likely to form by loop extrusion compared to TADs in cliques. We speculate that this is caused by the loop extrusion process requiring an accessible genome region where multiple interactions with other TADs are generally disfavored. Loop extrusion itself could also actively counteract long-range interactions. The apparent depletion in loop extrusion for singleton TADs could be explained by these TADs being less interactive and thus less associated with active genes. It has also been shown that TADs emerging from loop extrusion display a nested structure [14, 33] which could appear as binary-interacting TADs in our analyses.

### Relationship between TAD cliques and compartments

**Fig. 4:**
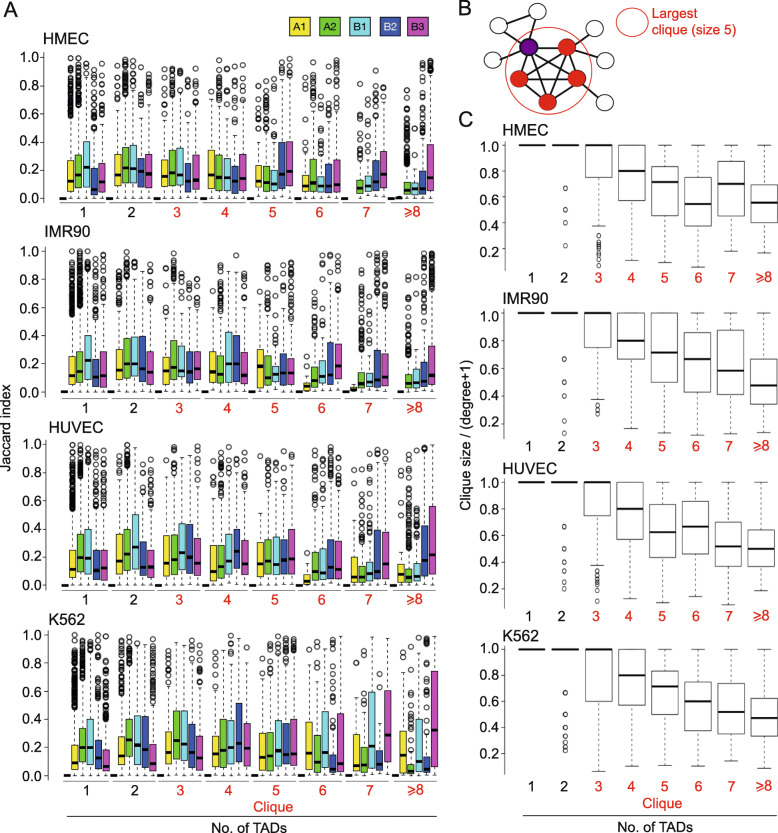
TADs in large cliques interact with a large number of TADs also outside the clique. **(A)** Overlap between singleton TADs, binary-interacting TADs and TADs in cliques (of indicated size) with A and B compartment subtypes. **(B)** Concept of ‘degree’ of TAD interactions. A given TAD (purple node) can belong to a clique of, here, size 3 (containing two other TADs [white nodes]) and a clique of size 5 (red nodes); the latter is the ‘maximal clique size’ (see main text). The total number of interactions involving the purple TAD (i.e. the TAD ‘degree’) is 7 and is materialized by 7 edges. In this example, the ratio of (clique size / (degree + 1)) is 5/(7 + 1) = 0.625 (see panel C). **(C)** Ratios of (clique size / (degree + 1)) for TADs identified as singletons, binary interacting and in cliques. The graphs consistently show that the larger the clique size, the lower the ratio, i.e. the greater the number of inter-TAD interactions a TAD engages in *outside* the clique. Note that for singleton TADs, this ratio is (trivially) always 1

Eigenvector analysis of high-resolution Hi-C data has been used to determine regions with a genomic size similar to TADs that segregate into six different subcompartments [3]. These have been shown to correspond to distinct types of active (subcompartment A1 and A2) and inactive (subcompartments B1-B4) regions of the genome. While both A1 and A2 are gene-dense and contain active histone marks, they complete DNA replication at the first half and middle of S-phase, respectively. Subcompartment B1 harbors H3K27me3 and is associated with facultative heterochromatin, while B2 and B3 are associated with LADs and constitutive heterochromatin (H3K9me2/me3). Unlike B2, B3 is depleted of association with the nucleolus [3]. The clique pattern of TAD-TAD interactions suggests a relationship with these subcompartments: we hypothesized that TADs in cliques behave as small, individual compartments, suggesting localized compartmentalization as a separate mechanism of TAD formation. To examine this possibility, we determined the overlap of subcompartment segments to TADs in cliques. Using the Jaccard index (JI) as a measure of the relative overlap between each TAD and its overlapping subcompartment(s), we found only a limited correspondence between these (median JI 0.1–0.3), irrespective of subcompartment type and cell type (Fig. 4 A). Notwithstanding, for all cell types except K562, A1 subcompartment overlap diminishes as TAD clique size increases (Fig. 4 A). The minor differences seen in subcompartment associations in K562 cells could speculatively be related to their cancer origin. For all cell types, overlap with B2 and B3 subcompartments tends to increase for larger clique sizes (Fig. 4 A). Notably, subcompartment B1 (facultative heterochromatin) shows a weak opposite trend, possibly explaining why singleton TADs are apparently less implicated in loop extrusion, since these would be less associated with active (and thus interactive) genes. We conclude from these observations that TAD cliques are distinct from previously annotated subcompartments.

To further understand the interaction patterns of TADs, we explored the relationship between TAD-TAD interactions and clique size, as this could shed light on whether TAD cliques might constitute an exclusive mode of regionalization of the genome rather than highly interacting compartments. More explicitly, we examined the relationship between the total number of TADs a given TAD interacts with and the size of the largest clique this TAD belongs to (Fig. 4B). Figure 4 C shows the ratio of (largest) clique size to the total number (‘degree’) of interactions of each TAD, for increasing clique sizes; this reflects how many of each TAD’s interactions are accounted for by their interactions in cliques. Consistently across cell types, we find that TADs in larger cliques tend to interact with a greater number of other TADs also outside the clique, resulting in lower clique size / interaction degree ratios (Fig. 4 C). We speculate that this may result from heterochromatin being more compact and interacting more closely with other heterochromatin regions, further supporting a view of preferred homotypic chromatin associations [8, 17, 32, 34]. In contrast, the lower density of inter-TAD interactions, manifested by high ratios involving TAD singletons, TAD pairs or small cliques of 3–4 TADs (Fig. 4 C) reflects more open chromatin configurations which are less interactive, except within TADs or with neighboring TADs (see e.g. Fig. 3 A).

To investigate how the configuration of TAD cliques may differ between cell types, we calculated all closest pairs of TAD cliques between HUVEC and all TAD cliques in K562, IMR90 and HMEC cells, using the Jaccard Index (JI). We then clustered the resulting matrix using k-means clustering to identify sets of cliques with similar (and different) TAD connectivity across cell types (Additional file 1, Fig. S10A). Using this approach, we identified 174 TAD cliques with a conserved connectivity (cluster 5; JI = 0.60–0.74), and 90 TAD cliques with a HMEC-specific connectivity (cluster 6; JI = 0.20–0.25), in addition to other clusters with partially shared characteristics (Additional file 1, Fig. S10B-D).

## Discussion

We report a genomic assessment of TADs in cliques, large multi-TAD assemblies detected from ensemble Hi-C data. Our results suggest that a subset of TADs serves regulatory function through the formation of long-range interactions, yet the definition of TADs has recently been challenged [11]. We also note that the nature of Hi-C contact domains is not fully understood. For example, Rowley et al. [35] report that approximately 25 % of TAD boundaries cannot be explained by extrusion or compartmentalization processes. The TADs in cliques reported here are characterized by being larger and less dense than typical TADs, and with a depletion of convergent CTCF motifs at their boundaries. This clearly suggests that chromatin loop extrusion cannot explain the formation of these TADs. Due to their large size, TADs in large cliques also do not fit the definition of compartment domains, which are typically smaller than TADs [13, 35]. The depletion of convergent CTCF motifs in TADs in cliques could also explain and support proposed models in which compartmentalization forces counteract loop extrusion [10].

Our results also indicate that TADs in larger cliques tend to be increasingly engaged in interactions also outside their clique. Thus, large cliques and interacting domains could constitute large heterochromatin assemblies reminiscent of gene-poor and peripheral ‘chromosome territory arrangements’ reported in several human cell types [36]. Our findings suggest that TAD cliques are embedded in a compact, yet interactive chromatin environment, and that processes shaping these domains may be different from those promoting compartmentalization. Microphase separation has been suggested to drive chromatin compartmentalization by facilitating attraction between homotypic domains [10, 37, 38]. Given the previously reported heterochromatic nature of TAD cliques, the association of large cliques with the nuclear lamina, and their localization at the nuclear periphery [21], heterochromatin tethering factors such as CBX5/HP1α [39], and nuclear envelope-associated heterochromatin anchoring proteins such as lamin A/C or lamin B receptor [40], could be involved in facilitating the formation or maintenance of TAD cliques. Knockdown of these factors in combination with Hi-C analysis and TAD clique identification could elucidate this further.

Moreover, we cannot rule out that the nuclear lamina (and nuclear periphery) itself provides an environment facilitating microphase separation of these domains. The inverted chromatin organization in rod cell nuclei of nocturnal mammals could provide an interesting model system to explore this further [41]. Recent chromatin modeling approaches have indeed suggested that heterochromatin-lamina interactions affect the interactivity of domains, and constitute a distinct force shaping nuclear organization [42]. It is also noteworthy that modeling approaches aiming to reconstitut the conventional (and inverted) center-periphery radial organization of mammalian nuclei critically require a separate force involving lamina-heterochromatin interactions [43].

Association of TAD cliques with LINE elements also supports recent evidence for retrotransposons shaping global genome architecture [31]. Intriguingly, this work shows homotypic clustering of LINE and SINE elements in the periphery and center of the nucleus, respectively, and points to transcription of some of these elements as a critical factor in establishing genome compartmentalization during embryogenesis [31]. Additional studies are needed to elucidate the potential relationship between TAD cliques and genome-wide LINE/SINE clustering in the nucleus.

In a recently suggested classification of Hi-C domains, TADs in TAD cliques would probably be classified as type-3 ‘compartment domain only: un-nested no-corner-dot compartment domain’ [14]. The large genomic size and relatively lower interaction density of these TADs compared to previously described compartment domains could however be indicative of a separate formation process.

We have relied on the Armatus TAD caller [23] for the delineation of TADs. This choice was based on testing a range of TAD callers and selecting the one that provided the most reproducible and visually pronounced TADs. It is however inevitable that some of the called TADs may be less well-defined using this algorithm. Thus, we cannot rule out that cliques between chromatin regions not readily identified as TADs also exist. TADs are the result of statistical aggregations of contacts in a cell population [11], so how TAD cliques appear in single cells remains to be investigated. In a first attempt to address this issue, we have reported that subsets of TADs in cliques identified in ensemble Hi-C matrices also show preferential association in single-cell Hi-C data [21]. Fluorescence *in situ* hybridization imaging of single cell nuclei also points to closer spatial proximity of TADs in cliques relative to TADs outside cliques [21]. Even if we have taken a TAD-based approach, our findings do not rule out that compartment domains not identified as TAD cliques serve important regulatory functions. Other complementary graph-based approaches could also be applied to further investigate TAD cliques. These include hierarchical community-detection approaches applied at even higher organizational levels to potentially detect long-range TAD interactions involved in cliques [18].

We find that binary interacting TADs, unlike singleton TADs, are the most enriched in convergent CTCF motifs. The explanation for this could be that binary interacting TADs are indicative of a nested TAD structure. These nested TAD structures have been shown to often be found for domains caused by loop-extrusion processes [14]. Also, core-periphery topology structures within TADs have recently been demonstrated to be relevant features for subsets of TADs [20]. Additional investigations linking intra-TAD topology to TAD cliques are needed to elucidate this further.

## Conclusions

We find TAD cliques across different cell types, suggesting that TAD cliques are general phenomena not only linked to cell differentiation. In this regard, TAD cliques constitute an interesting and important chromatin feature for further study, since they link local interaction patterns (i.e. TADs and compartment domains) to higher order organization (i.e. compartments and LADs). A deeper characterization of TAD cliques across cell and tissue types might further elucidate these relationships. Also, single-cell analysis, including high-throughput imaging, might reveal whether TAD cliques result from an aggregation of interactions across cells, or exist within single cells. Taken together, our results shed further light on the increasingly complex picture of multiscale chromatin organization.

## Methods

### Hi-C data

To uniformly process all Hi-C data used in this study, raw data were downloaded from ENCODE [22] and processed using the HiC-Pro pipeline [44] (https://github.com/nservant/HiC-Pro). First, the paired-end sequences were mapped to the hg38 reference genome using Bowtie2 [45] with default parameters preset in HiC-Pro configuration file. Unmapped, multi-mapped, singletons and low map quality reads were removed and only uniquely mapped reads were used for binning, normalizing and generating Hi-C matrices. The pipeline produced raw and normalized interaction frequency matrices. For further analyses, 5-kb and 50-kb resolution raw matrices were used for all cell lines. We used the hicpro2juicebox.sh script from HiC-Pro to convert matrices into .hic files for visualization with Juicebox [46] (https://github.com/theaidenlab/juicebox).

### TAD calling

TADs were called using Armatus v2.1.0 [23] (https://github.com/kingsfordgroup/armatus) using a gamma of 1.2 for all cell lines. Genomic regions not defined as TADs by Armatus were nevertheless included to ensure full genome segmentation. TADs were visualized using Juicebox (Fig. 1 A).

### Identification of TAD-TAD interactions

TAD-TAD interactions were identified using the NCHG (Non-central Hypergeometric model) tool [24]. Hi-C contacts were aggregated to generate TAD-TAD interaction matrices for each cell line. NCHG was used to calculate P values for each TAD pair. For each pair, this model takes into account the total number of interactions that the two TADs engage in, the genomic distance between them, and the total number of contacts for the chromosome. In effect, in addition to their inter-TAD distance, this model adjusts for factors depending on the variable size of the TADs and possible contact differences due to experimental conditions (e.g. enzyme accessibility or other factors). On these P values, we performed multiple testing correction with a false discovery rate (FDR) < 1 % using the Benjamini-Hochberg method. The resulting significant interactions were filtered by requiring a five-fold enrichment of observed over expected contacts based on genomic distance.

The network configuration of TAD-TAD interactions (Fig. 1B) was generated using the igraph R package [47] (https://github.com/igraph/rigraph). The igraph layout was made using the 131 TADs identified in chromosome 18 of IMR90 cells. We used the ‘graphopt’ algorithm setting the charge parameter to 0.03 while the remaining parameters were left as default. Each node was colored-coded based on the degree of interactions.

### TAD clique calling

As we reported earlier [21], significant TAD-TAD interactions were represented as a graph using the NetworkX Python library (http://networkx.github.io/). In the graph, TADs are represented by nodes and significant interactions between them are represented by edges. Maximal TAD clique sizes were calculated using the Bron-Kerbosch algorithm [48]. Maximal clique size (*k*) was assigned to each TAD, where *k* is the size of the largest TAD clique to which the TAD belongs to.

### Gene ontology analyses

Gene ontology enrichment analysis and statistics were performed using g:Profiler [49] (https://biit.cs.ut.ee/gprofiler/).

### TAD clique clustering

For each TAD clique in HMEC, we identified the TAD clique with maximal similarity (based on the Jaccard index [JI]) in all the other cell lines (K562, IMR90, HUVEC). From this we computed a matrix containing all maximal JI values, centered at HMEC, for all pairs of TAD cliques (Additional file 1, Fig. S9). We then clustered the resulting matrix using k-means clustering (k = 8) to identify sets of cliques with similar (and different) TAD-connectivity across cell-types.

### Repeat analysis

The repeat mask file for the hg38 genome assembly was downloaded from the UCSC genome browser [50] (http://hgdownload.cse.ucsc.edu/goldenpath/hg38/database/rmsk.txt.gz). From the repeat mask file, the following repeats were selected for further analysis: LINE, SINE, LTR, retrotransposons, rRNA, satellite, simple and DNA. The repeat contents for each TAD were calculated using the bedtools coverage option [51] and plots generated using the ggplot2 R package.

### Aggregated TADs

Intra-TAD interaction frequencies for each TAD in IMR90 cells at 5 kb resolution was extracted from the Hi-C matrix. As the genomic length of TADs differs, so do the sizes of intra-TAD interaction frequency matrices. Therefore, all TADs were resized to a 25 × 25 matrix using the ‘nearest’ algorithm from the OpenImageR R package (https://github.com/mlampros/OpenImageR). The element-wise mean was calculated for all TADs of a given category (based on clique size) to produce the mean matrix for that category.

### CTCF motif orientation analysis

Processed CTCF peak files in NarrowPeak format for all cell lines were downloaded from ENCODE [22]. The GimmeMotif [52], a transcription factor analysis tool, was used to call all motifs from the peak files using the ‘scan’ option passing the ‘JASPAR2020_vertebrates’ PFM file. From the resulting bed file, CTCF peaks were extracted with information on the orientation of CTCF binding. Python and R scripts were used to calculate the CTCF orientations at TAD boundaries.

### Scripting

All scripts for data analyses in this study were written using R, Python and Bash. The scripts can be found on GitHub (https://github.com/tharvesh/paper3).

## Supplementary information

Additional file 1: **Table S1.** ENCODE data accession numbers. **Table S2.** Numbers of TADs in cliques and non-cliques. **Table S3.** Statistics on enrichment of convergent CTCF sites in TADs as a function of clique size. **Table S4.** Statistics on enrichment of convergent CTCF sites in TADs in B compartments, as a function of clique size. **Figure S1.** TAD-TAD interactions and chromatin marks. **(A)** Example of a TAD clique (size 5) in chromosome 18. Middle panel shows the Hi-C data with the 5 TADs in the clique highlighted in black squares. Corresponding relative enrichments of epigenetic marks are shown in the top/left panels. **(B)** Enlarged region highlighting two individual TADs (on the diagonal) and their pairwise interaction (top right square). **Figure S2.** Gene ontology terms enriched in HMEC TAD cliques. **Figure S3.** Gene ontology terms enriched in IMR90 TAD cliques. **Figure S4.** Gene ontology terms enriched in K562 TAD cliques. **Figure S5.** Gene ontology terms enriched in HUVEC TAD cliques. **Figure S6.** Gene ontology terms enriched in all TAD cliques combined across the four cell types examined in this study. **Figure S7.** Genomic characterization of TADs in cliques and outside cliques in B compartments only. **(A)** Number of TADs (Armatus) in cliques and outside cliques in B compartments. **(B)** Distribution of gene expression levels in TADs in cliques and outside cliques in B compartments. P values (K-S tests): HMEC *P* = 1.2e-05; IMR90 *P* = 0.07; HUVEC *P* = 2.4e-04; K562 *P* < 2.2e-16. **(C)** Proportion of TAD coverage by indicated repeat classes in cliques and outside cliques in B compartments. **Figure S8.** Enrichment (in % of base pairs) of non-mappable repetitive regions (from ENCODE) (y axis) in TADs belonging to different TAD clique size categories (x axis). **Figure S9.** Proportions of convergent CTCF motifs for TADs in cliques in B compartments only. Percentage of convergent CTCF motifs at the boundaries of TADs in B compartments categorized as shown. Horizontal bar represents the average percentage of convergent CTCF motifs in all TADs in B compartments. *Binomial test; *P < 0.05; see **Table S4** for statistics. **Figure S10.** Clustering analysis of TAD cliques across cell types. **(A)** Clustered heatmap showing the maximal Jaccard Index (JI) of comparisons of all sets of TAD cliques in HMEC compared to K562 (first row in heatmap), IMR90 (second row) and HUVEC (third row). The heatmap was clustered using k-means (k = 8). Numbers on top indicate cluster IDs of all 8 clusters with number of TAD cliques belonging to each cluster shown in parenthesis. Green box highlights cluster 5 which contains TAD cliques with a similar configuration in all cell types; blue box highlights cluster 6 which contains TAD cliques with a dissimilar configuration across cell types. **(B)** Browser view example of a TAD clique of size 6 (genomic positions indicated by the bottom blue segments) on chromosome 8. Clique sizes are shown above each TAD and indicated using red shades. **(C)** Average JI for the 8 clusters. **(D)** Browser view example of a clique of size 6 (positions indicated by the bottom blue segments) on chromosome 6. TAD clique sizes are shown above each TAD and indicated using red color shades.

## Data Availability

The datasets supporting the conclusions of this article are available in the ENCODE repository at https://www.encodeproject.org/ using accession numbers provided in Additional file 1, Table S1.

## Abbreviations

JI: Jaccard index
LAD: lamina-associated domain
LINE: long interspersed element
SINE: long interspersed element
TAD: topologically-associated domain
