# Supplementary material for: TAD cliques predict key features of chromatin organization
Source: BMC Genomics. 2021 Jul 3;22:499. doi: 10.1186/s12864-021-07815-8 (PMC8254932; doi:10.1186/s12864-021-07815-8)
Supplement: Supplementary file 1 — Table S1. ENCODE data accession numbers. Table S2. Numbers of TADs in cliques and non-cliques. Table S3. Statistics on enrichment of convergent CTCF sites in TADs as a function of clique size. Table S4. Statistics on enrichment of convergent CTCF sites in TADs in B compartments, as a function of clique size. Figure S1. TAD-TAD interactions and chromatin marks. (A) Example of a TAD clique (size 5) in chromosome 18. Middle panel shows the Hi-C data with the 5 TADs in the clique highlighted in black squares. Corresponding relative enrichments of epigenetic marks are shown in the top/left panels. (B) Enlarged region highlighting two individual TADs (on the diagonal) and their pairwise interaction (top right square). Figure S2. Gene ontology terms enriched in HMEC TAD cliques. Figure S3. Gene ontology terms enriched in IMR90 TAD cliques. Figure S4. Gene ontology terms enriched in K562 TAD cliques. Figure S5. Gene ontology terms enriched in HUVEC TAD cliques. Figure S6. Gene ontology terms enriched in all TAD cliques combined across the four cell types examined in this study. Figure S7. Genomic characterization of TADs in cliques and outside cliques in B compartments only. (A) Number of TADs (Armatus) in cliques and outside cliques in B compartments. (B) Distribution of gene expression levels in TADs in cliques and outside cliques in B compartments. P values (K-S tests): HMEC P = 1.2e-05; IMR90 P = 0.07; HUVEC P = 2.4e-04; K562 P < 2.2e-16. (C) Proportion of TAD coverage by indicated repeat classes in cliques and outside cliques in B compartments. Figure S8. Enrichment (in % of base pairs) of non-mappable repetitive regions (from ENCODE) (y axis) in TADs belonging to different TAD clique size categories (x axis). Figure S9. Proportions of convergent CTCF motifs for TADs in cliques in B compartments only. Percentage of convergent CTCF motifs at the boundaries of TADs in B compartments categorized as shown. Horizontal bar represents the average percentage of convergent CTCF [file 12864_2021_7815_MOESM1_ESM.pdf]

TAD cliques predict key features of chromatin organization

Tharvesh M. Liyakat Ali, Annaël Brunet, Philippe Collas* and Jonas Paulsen*

*Correspondence.

Additional file

Additional file 1. A PDF containing 4 supplemental tables and 10 supplemental figures.

Additional file 1

Table S1. ENCODE data accession numbers

Data type	IMR90	HMEC	HUVEC	K562
**Hi-C raw data**	SRR1658672	SRR1658680	SRR1658709	SRR1658693
	SRR1658673	SRR1658681	SRR1658710	SRR1658694
	SRR1658674	SRR1658682	SRR1658711	SRR1658695
	SRR1658675	SRR1658683	SRR1658712	SRR1658696
	SRR1658676	SRR1658684	SRR1658713	SRR1658697
	SRR1658677	SRR1658685	SRR1658714	SRR1658698
	SRR1658678			SRR1658699
				SRR1658700
				SRR1658701
				SRR1658702
**RNA-seq data**	ENCFF244VME	ENCFF526QYF	ENCFF238WEU	ENCFF104VTJ
**ChIP-seq data**	ENCFF307XFM	ENCFF641CHY	ENCFF536OTV	ENCFF873LHT

Table S2. Numbers of TADs in cliques and non-cliques

	Number of TADs		
**Cell type**	**Clique**	**Non-clique**	**Total**
HMEC	1486	4522	6008
IMR90	1554	4095	5649
HUVEC	1189	4313	5502
K562	1488	4332	5820

Table S3. Statistics on enrichment of convergent CTCF sites in TADs as a function of clique size

Clique category	Confident1	Confident2	TADs with conv. CTCF motifs	No. TADs in category	% TADs with conv. CTCF in genome	P-value
**IMR90**						
**==2**	0.1835	0.2405	173	820	0.1871	0.0809
**>=2**	0.1866	0.2194	481	2374	0.1871	0.0547
**>=3**	0.1786	0.2189	308	1554	0.1871	0.2688
**>=4**	0.1669	0.2135	214	1130	0.1871	0.8488
**>=5**	0.1560	0.2084	157	867	0.1871	0.6951
**>=6**	0.1443	0.2021	118	687	0.1871	0.3278
**>=7**	0.1321	0.1986	82	502	0.1871	0.1880
**>=8**	0.1008	0.1743	48	357	0.1871	0.0098
**>=9**	0.0756	0.1646	25	218	0.1871	0.0053
**>=10**	0.0538	0.1508	15	159	0.1871	0.0021
**HMEC**						
**==2**	0.2182	0.2757	220	894	0.2006	0.0010
**>=2**	0.2016	0.2352	519	2380	0.2006	0.0358
**>=3**	0.1811	0.2225	299	1486	0.2006	0.9484
**>=4**	0.1744	0.2239	205	1034	0.2006	0.8766
**>=5**	0.1433	0.1978	129	762	0.2006	0.0299
**>=6**	0.1256	0.1878	85	549	0.2006	0.0065
**>=7**	0.1218	0.1997	57	361	0.2006	0.0417
**>=8**	0.1100	0.1982	41	273	0.2006	0.0409
**>=9**	0.0904	0.1921	26	192	0.2006	0.0239
**>=10**	0.0788	0.2091	16	119	0.2006	0.0850
**HUVEC**						
**==2**	0.1749	0.2325	158	780	0.1785	0.0835
**>=2**	0.1733	0.2085	375	1969	0.1785	0.1667
**>=3**	0.1609	0.2057	217	1189	0.1785	0.7050
**>=4**	0.1457	0.1975	144	845	0.1785	0.5595
**>=5**	0.1399	0.1991	108	643	0.1785	0.5365
**>=6**	0.1312	0.2000	77	471	0.1785	0.4340
**>=7**	0.1202	0.1998	54	344	0.1785	0.3244
**>=8**	0.1058	0.2140	29	188	0.1785	0.4460
**>=9**	0.0998	0.2400	19	118	0.1785	0.7185
**>=10**	0.0537	0.2490	7	54	0.1785	0.4760
**K562**						
**==2**	0.1763	0.2271	200	996	0.1720	0.0186
**>=2**	0.1767	0.2081	477	2484	0.1720	0.0091
**>=3**	0.1667	0.2069	277	1488	0.1720	0.1491
**>=4**	0.1491	0.1963	175	1019	0.1720	1.0000
**>=5**	0.1354	0.1885	124	772	0.1720	0.4453
**>=6**	0.1259	0.1878	86	555	0.1720	0.3113
**>=7**	0.1040	0.1748	53	388	0.1720	0.0691
**>=8**	0.0651	0.1451	24	240	0.1720	0.0026
**>=9**	0.0393	0.1344	11	142	0.1720	0.0017
**>=10**	0.0247	0.1656	5	67	0.1720	0.0343

Table S4. Statistics on enrichment of convergent CTCF sites in TADs in B compartments, as a function of clique size

Clique catego	Confident1	Confident2	ADs with conv. CTCF motio	TADs in catego	ADs with conv. CTCF in geno	P-value
**IMR90**						
==1	0.0777	0.1410	42	395	0.1478	0.0191
==2	0.0937	0.2051	24	168	0.1478	1.0000
>=2	0.1442	0.2023	117	681	0.1478	0.0838
>=3	0.1489	0.2174	93	513	0.1478	0.0344
>=4	0.1390	0.2179	67	381	0.1478	0.1292
>=5	0.1408	0.2302	56	307	0.1478	0.0911
>=6	0.1392	0.2384	47	254	0.1478	0.1107
>=7	0.1097	0.2188	30	189	0.1478	0.6815
>=8	0.0804	0.2011	18	136	0.1478	0.7170
>=9	0.0452	0.1758	9	93	0.1478	0.1894
>=10	0.0156	0.1380	4	71	0.1478	0.0284
**HMEC**						
==1	0.0950	0.1522	65	535	0.1756	0.0008
==2	0.1877	0.3100	50	204	0.1756	0.0126
>=2	0.1841	0.2423	168	792	0.1756	0.0088
>=3	0.1690	0.2354	118	588	0.1756	0.1158
>=4	0.1701	0.2445	98	477	0.1756	0.0918
>=5	0.1599	0.2417	77	388	0.1756	0.2302
>=6	0.1506	0.2423	59	305	0.1756	0.4079
>=7	0.1395	0.2431	44	235	0.1756	0.6078
>=8	0.1195	0.2369	30	174	0.1756	1.0000
>=9	0.0989	0.2327	20	127	0.1756	0.7261
>=10	0.0790	0.2445	12	81	0.1756	0.6607
**HUVEC**						
==1	0.0680	0.1268	39	413	0.1380	0.0100
==2	0.0751	0.1939	17	135	0.1380	0.8028
>=2	0.1420	0.2109	86	493	0.1380	0.0221
>=3	0.1532	0.2375	69	358	0.1380	0.0044
>=4	0.1420	0.2353	53	286	0.1380	0.0254
>=5	0.1249	0.2236	41	241	0.1380	0.1604
>=6	0.1144	0.2226	32	196	0.1380	0.3003
>=7	0.0913	0.2114	21	146	0.1380	0.8104
>=8	0.0800	0.2474	12	80	0.1380	0.7453
>=9	0.0604	0.2498	8	59	0.1380	1
>=10	0.0013	0.2603	1	19	0.1380	0.5025
**K562**						
==1	0.0843	0.1307	76	718	0.1259	0.1149
==2	0.1011	0.2202	24	156	0.1259	0.2781
>=2	0.1226	0.1826	87	577	0.1259	0.0785
>=3	0.1169	0.1874	63	421	0.1259	0.1418
>=4	0.1000	0.1761	45	334	0.1259	0.6205
>=5	0.0825	0.1632	32	270	0.1259	0.7834
>=6	0.0769	0.1700	24	204	0.1259	0.8327
>=7	0.0496	0.1451	14	157	0.1259	0.1860
>=8	0.0267	0.1302	7	107	0.1259	0.0583
>=9	0.0153	0.1362	4	72	0.1259	0.0755
>=10	0.0060	0.1653	2	41	0.1259	0.1617

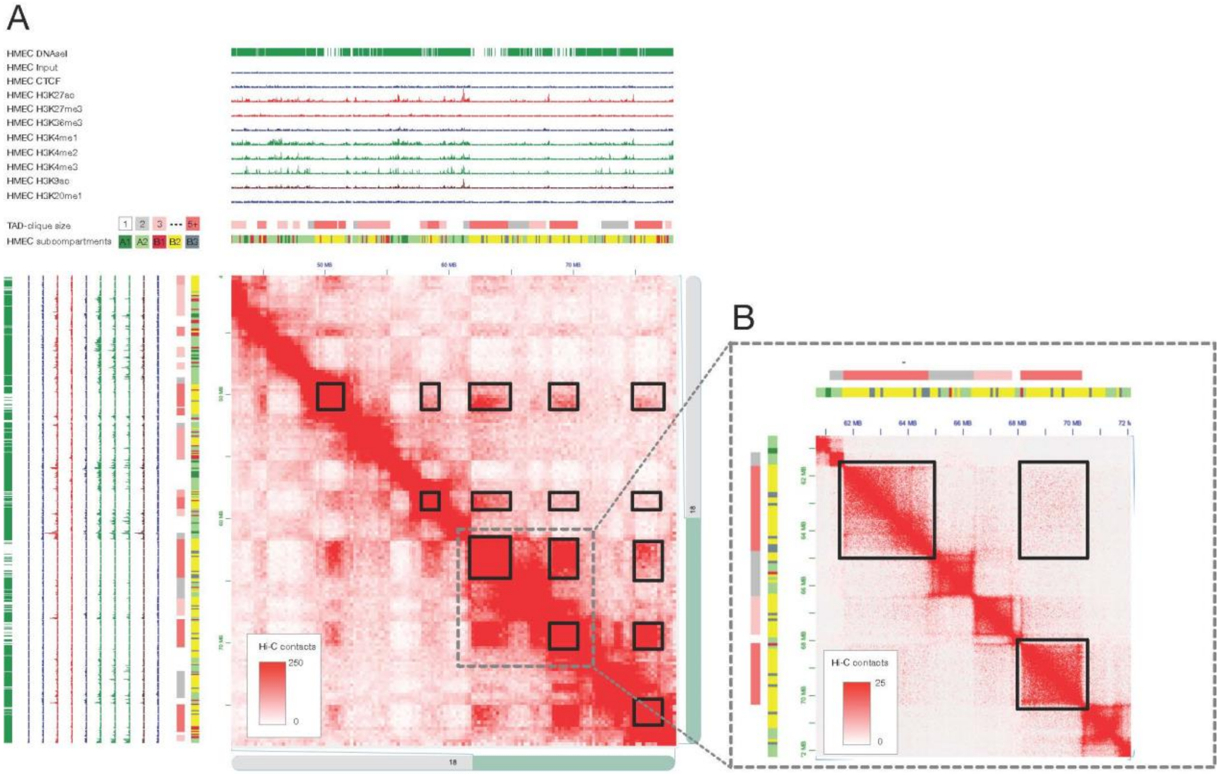

Figure S1. TAD-TAD interactions and chromatin marks. **(A)** Example of a TAD clique (size 5) in chromosome 18. Middle panel shows the Hi-C data with the 5 TADs in the clique highlighted in black squares. Corresponding relative enrichments of epigenetic marks are shown in the top/left panels. **(B)** Enlarged region highlighting two individual TADs (on the diagonal) and their pairwise interaction (top right square).

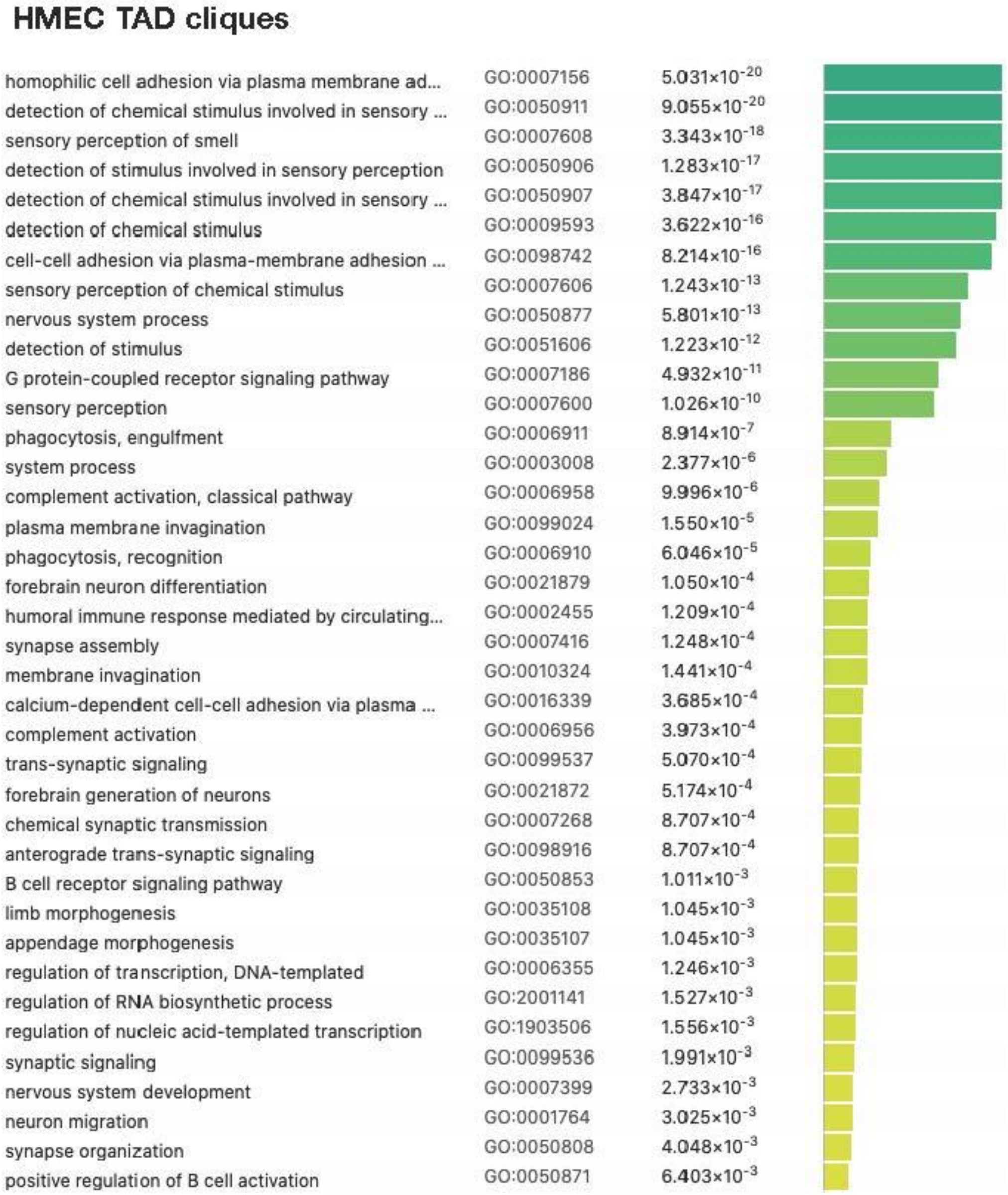

Figure S2. Gene ontology terms enriched in HMEC TAD cliques.

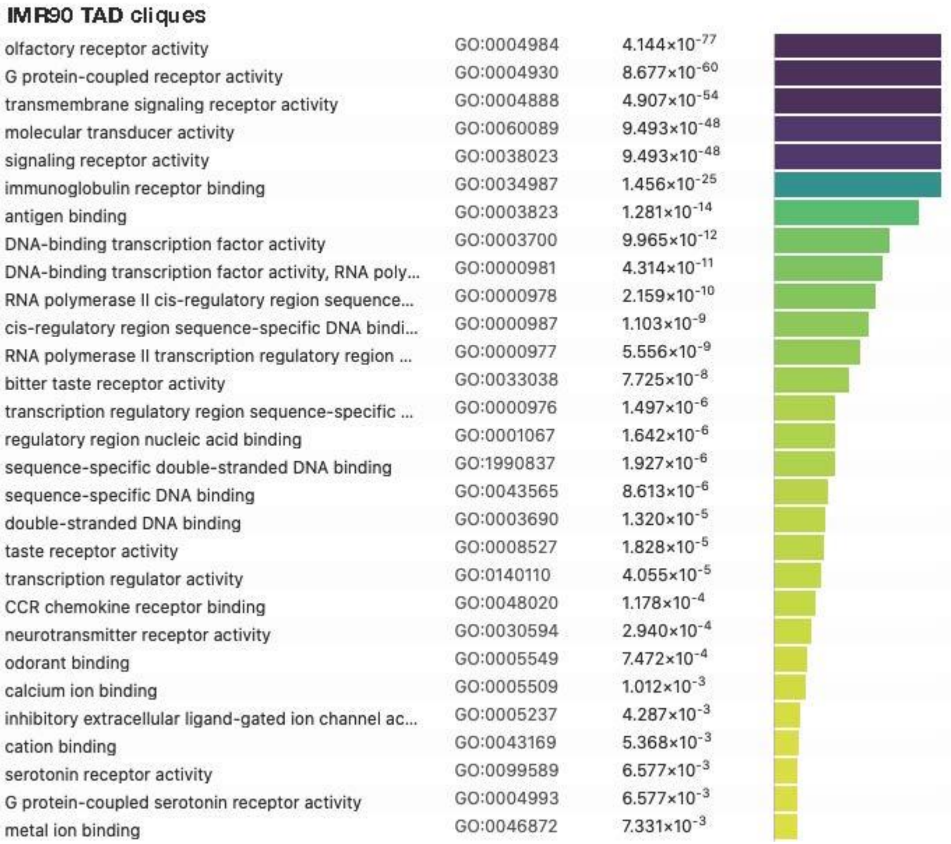

Figure S3. Gene ontology terms enriched in IMR90 TAD cliques.

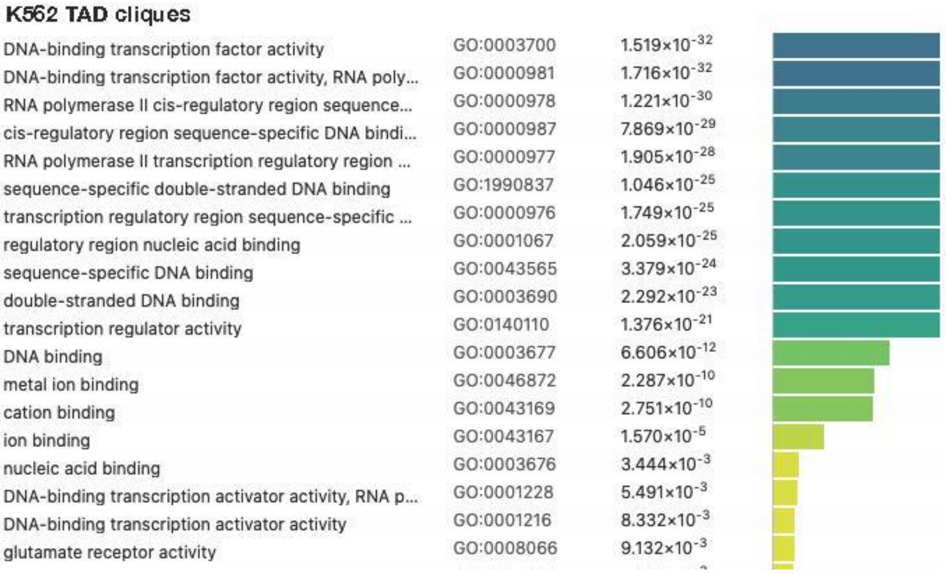

Figure S4. Gene ontology terms enriched in K562 TAD cliques.

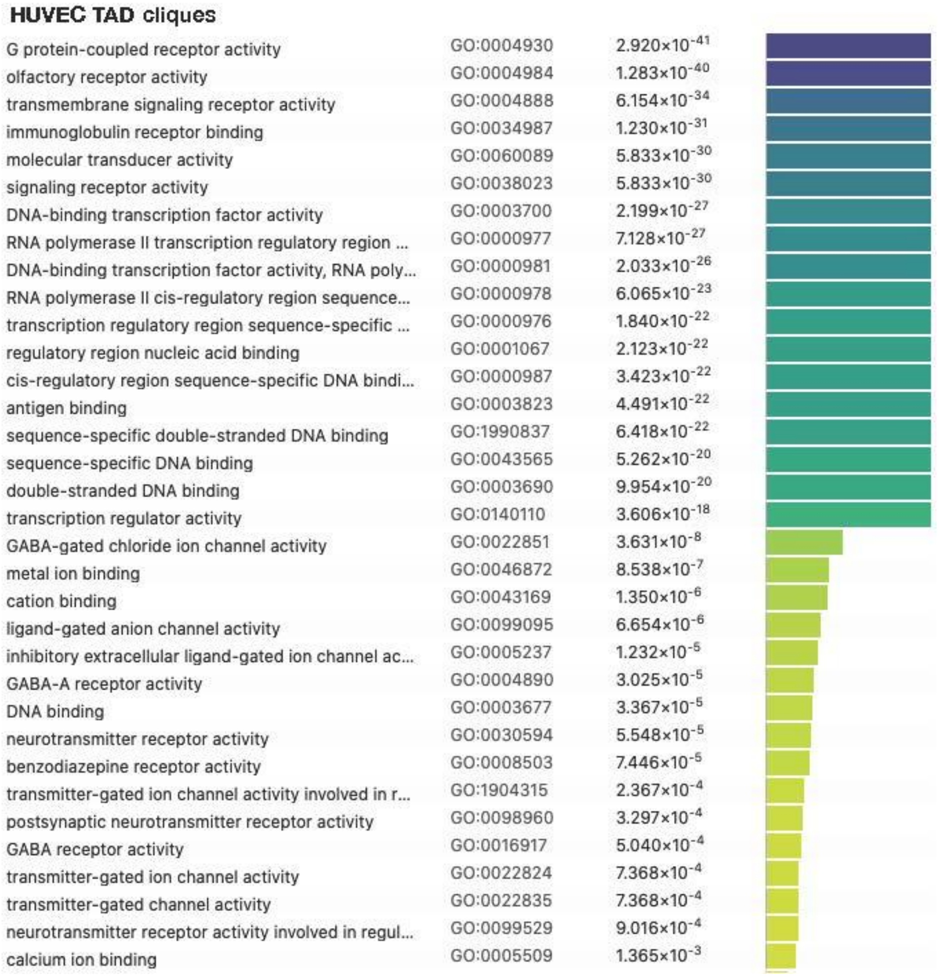

Figure S5. Gene ontology terms enriched in HUVEC TAD cliques.

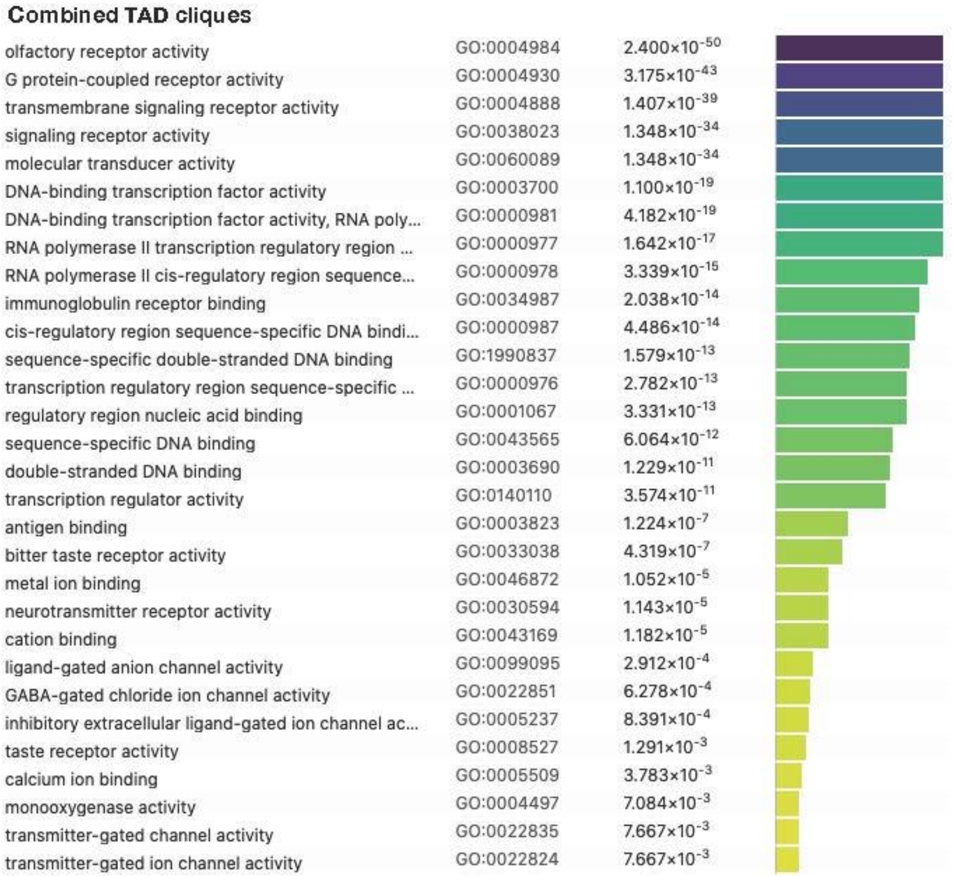

Figure S6. Gene ontology terms enriched in all TAD cliques combined across the four cell types examined in this study.

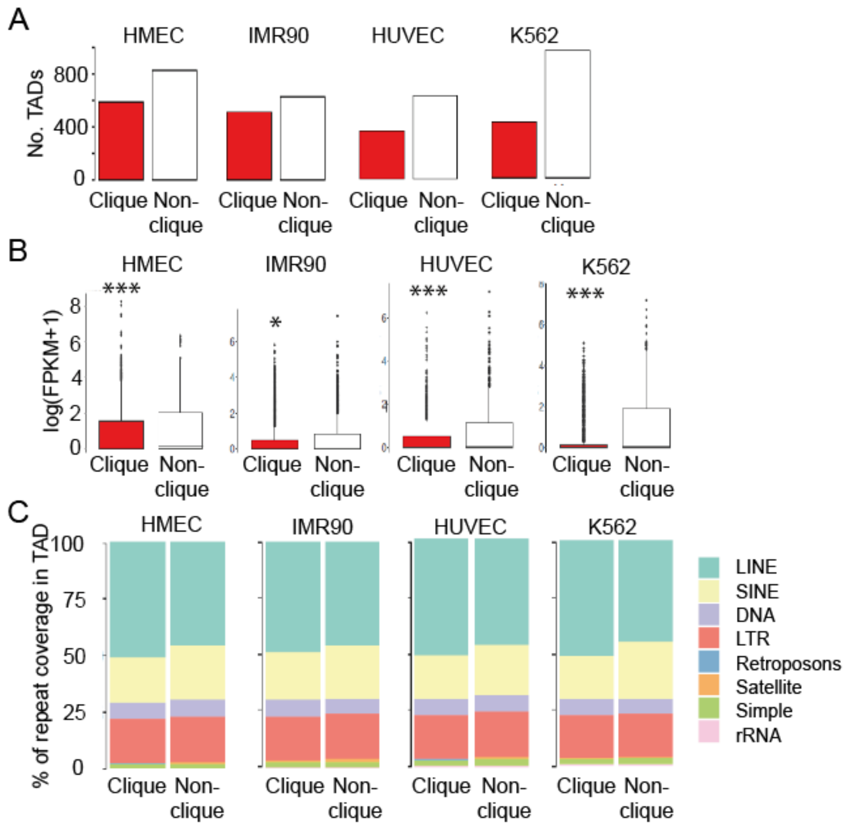

Figure S7. Genomic characterization of TADs in cliques and outside cliques in B compartments only. **(A)** Number of TADs (Armatus) in cliques and outside cliques in B compartments. **(B)** Distribution of gene expression levels in TADs in cliques and outside cliques in B compartments. P values (K-S tests): HMEC P = 1.2e-05; IMR90 P = 0.07; HUVEC P = 2.4e-04; K562 P < 2.2e-16. **(C)** Proportion of TAD coverage by indicated repeat classes in cliques and outside cliques in B compartments.

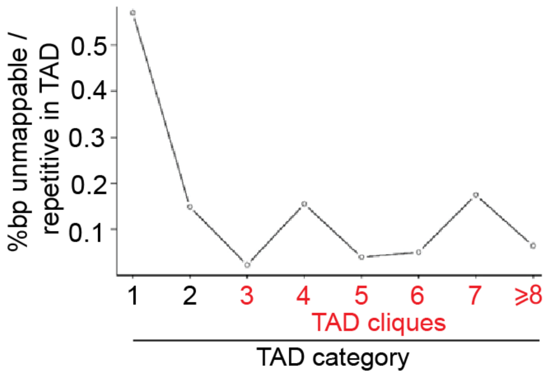

Figure S8. Enrichment (in % of base pairs) of non-mappable repetitive regions (from ENCODE) (y axis) in TADs belonging to different TAD clique size categories (x axis).

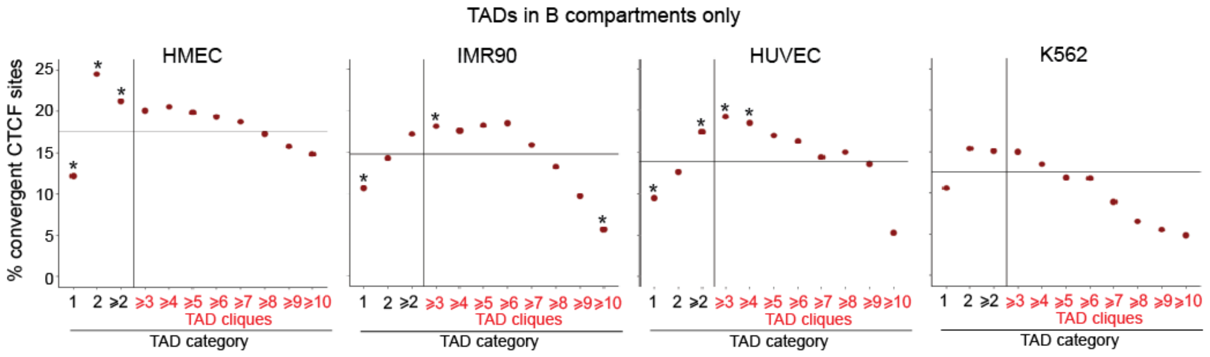

Figure S9. Proportions of convergent CTCF motifs for TADs in cliques in B compartments only. Percentage of convergent CTCF motifs at the boundaries of TADs in B compartments categorized as shown. Horizontal bar represents the average percentage of convergent CTCF motifs in all TADs in B compartments. *Binomial test; *P < 0.05; see **Table S4** for statistics.

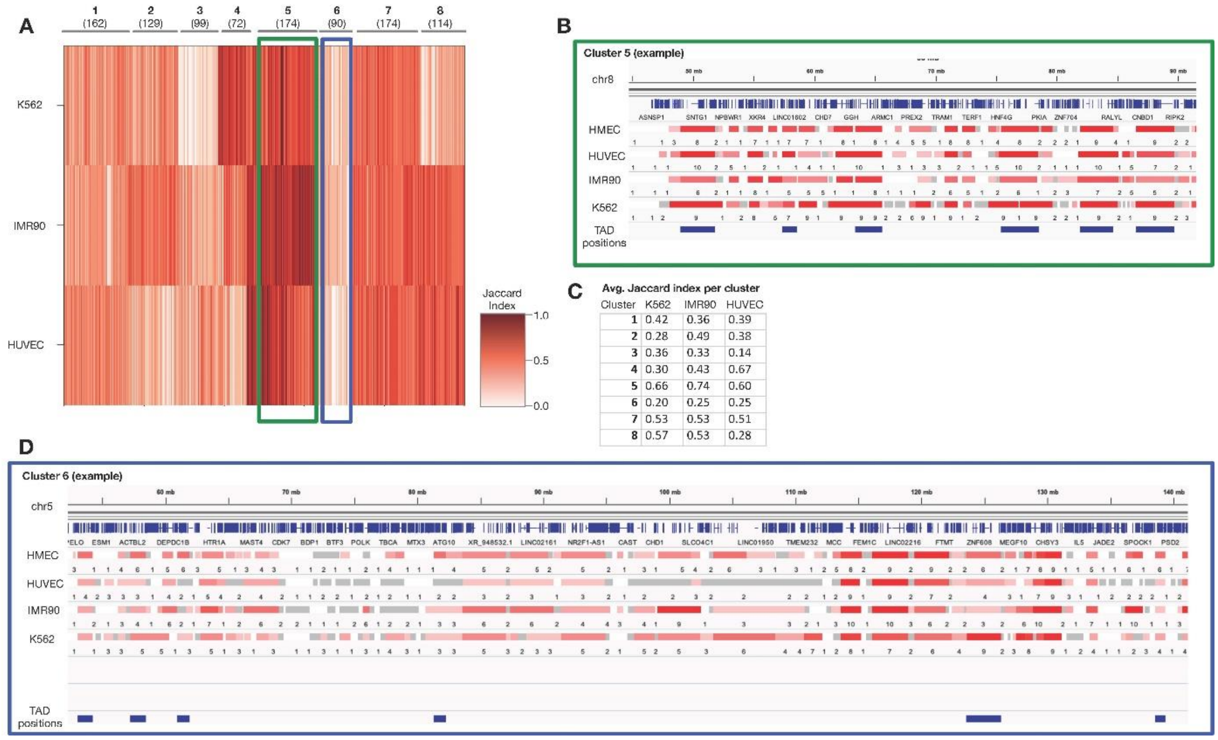

Cluster	K562	IMR90	HUVEC
1	0.42	0.36	0.39
2	0.28	0.49	0.38
3	0.36	0.33	0.14
4	0.30	0.43	0.67
5	0.66	0.74	0.60
6	0.20	0.25	0.25
7	0.53	0.53	0.51
8	0.57	0.53	0.28

Figure S10. Clustering analysis of TAD cliques across cell types. **(A)** Clustered heatmap showing the maximal Jaccard Index (JI) of comparisons of all sets of TAD cliques in HMEC compared to K562 (first row in heatmap), IMR90 (second row) and HUVEC (third row). The heatmap was clustered using k-means (k=8). Numbers on top indicate cluster IDs of all 8 clusters with number of TAD cliques belonging to each cluster shown in parenthesis. Green box highlights cluster 5 which contains TAD cliques with a similar configuration in all cell types; blue box highlights cluster 6 which contains TAD cliques with a dissimilar configuration across cell types. **(B)** Browser view example of a TAD clique of size 6 (genomic positions indicated by the bottom blue segments) on chromosome 8. Clique sizes are shown above each TAD and indicated using red shades. **(C)** Average JI for the 8 clusters. **(D)** Browser view example of a clique of size 6 (positions indicated by the bottom blue segments) on chromosome 6. TAD clique sizes are shown above each TAD and indicated using red color shades.
